# Myricetin improves endurance capacity and mitochondrial density by activating SIRT1 and PGC-1α

**DOI:** 10.1038/s41598-017-05303-2

**Published:** 2017-07-24

**Authors:** Hoe-Yune Jung, Dongyeop Lee, Hye Guk Ryu, Bo-Hwa Choi, Younghoon Go, Namgyu Lee, Dohyun Lee, Heehwa G. Son, Jongsu Jeon, Seong-Hoon Kim, Jong Hyuk Yoon, Seon-Min Park, Seung-Jae V. Lee, In-Kyu Lee, Kwan Yong Choi, Sung Ho Ryu, Kazunari Nohara, Seung-Hee Yoo, Zheng Chen, Kyong-Tai Kim

**Affiliations:** 1 Department of Integrative Biosciences & Biotechnology, Pohang University of Science and Technology(POSTECH), 77 Cheongam-Ro, Pohang, Gyeongbuk 37673 Republic of Korea; 2 Department of Life Sciences, Pohang University of Science and Technology(POSTECH), 77 Cheongam-Ro, Pohang, Gyeongbuk 37673 Republic of Korea; 3 Information Technology Convergence Engineering, Pohang University of Science and Technology(POSTECH), 77 Cheongam-Ro, Pohang, Gyeongbuk 37673 Republic of Korea; 4 School of Interdisciplinary Bioscience and Bioengineering, Pohang University of Science and Technology(POSTECH), 77 Cheongam-Ro, Pohang, Gyeongbuk 37673 Republic of Korea; 5 R&D Center, NovMetaPharma Co., Ltd., 394 Jigok-Ro, Pohang, Gyeongbuk 37668 Republic of Korea; 6 Advanced Bio Convergence Center, Pohang Technopark, 394 Jigok-Ro, Pohang, Gyeongbuk 37668 Republic of Korea; 7 Department of Veterinary Medicine, Kyungpook, National University, Daegu, 41566 Republic of Korea; 8 Leading-Edge Research Center for Drug Discovery and Development for Diabetes and Metabolic Disease, Kyungpook National University Hospital, Daegu, 41407 South Korea; 9 Department of Biochemistry and Molecular Biology, The University of Texas Health Science Center at Houston, 6431 Fannin St., Houston, TX 77030 USA; 10 Korean Medicine (KM) Application Center, Korea Institute of Oriental Medicine (KIOM), 41062 Daegu, Republic of Korea; 11 Department of Molecular, Cell and Cancer Biology, University of Massachusetts Medical School, Worcester, MA, 01605 USA

## Abstract

Robust mitochondrial respiration provides energy to support physical performance and physiological well-being, whereas mitochondrial malfunction is associated with various pathologies and reduced longevity. In the current study, we tested whether myricetin, a natural flavonol with diverse biological activities, may impact mitochondrial function and longevity. The mice were orally administered myricetin (50 mg/kg/day) for 3 weeks. Myricetin significantly potentiated aerobic capacity in mice, as evidenced by their increased running time and distance. The elevated mitochondrial function was associated with induction of genes for oxidative phosphorylation and mitochondrial biogenesis in metabolically active tissues. Importantly, myricetin treatment led to decreased PGC-1α acetylation through SIRT1 activation. Furthermore, myricetin significantly improved the healthspan and lifespan of wild-type, but not *Sir-2.1*-deficient, *C. elegans*. These results demonstrate that myricetin enhances mitochondrial activity, possibly by activating PGC-1α and SIRT1, to improve physical endurance, strongly suggesting myricetin as a mitochondria-activating agent.

## Introduction

Mitochondria are the primary site for cellular energy production, providing ATPs through aerobic respiration. Conversely, impaired mitochondrial function has been implicated in various diseases, particularly affecting metabolic, cardiovascular and nervous systems^1, 2^. For example, it has been well documented that metabolic and cardiovascular risk factors are correlated with low aerobic capacity and reduced muscle expression of genes required for oxidative phosphorylation (OXPHOS) and mitochondrial biogenesis^3^. One such gene encodes the peroxisome proliferator-activated receptor γ coactivator, PGC-1α^4^. PGC-1α functions as a coactivator for nuclear receptors and plays a predominant regulatory role in energy metabolism and mitochondrial biogenesis and respiration^5, 6^. Previous studies have revealed that PGC-1α controls muscle fiber-type switching^7^ and adaptive thermogenesis in the brown adipose tissue (BAT)^8^. Another group of key regulators capable of promoting mitochondriogenesis and enhancing respiration are the sirtuin family of deacetylases (SIRTs)^9^. In mammals, seven sirtuin genes have been identified (Sirt1–Sirt7)^10^. Among them, SIRT1 belongs to class III histone/protein deacetylases, and its dysregulation has been shown to be involved in aging, metabolic diseases and inflammation^11–13^. Under caloric restriction (CR), SIRT1 functions coordinately with PGC-1α to promote metabolic adaptation^14^. In particular, SIRT1 activates PGC-1α by deacetylation of lysine residues, thereby enhancing mitochondrial biogenesis^15^. The deacetylase activity of SIRT proteins is dependent on the intracellular NAD + content^16^.

Given the close association between aging and mitochondrial function, it is conceivable that a small-molecular activator of SIRT1 can display anti-aging efficacies. On the other hand, an age-related decline in mitochondrial function is thought to be a contributing factor to insulin resistance and cancers^1^. Interestingly, CR, a well-established anti-aging manipulation conserved in various organisms, is known to improve mitochondrial function^17^. Therefore, SIRT1-activating agents can impinge upon overlapping pathways that are also modified by CR and serve as potential therapeutic regimens for diseases of aging. In that regard, resveratrol, a well-known SIRT1 activator, has been shown to confer protection against metabolic disorders by activating mitochondrial respiration and biogenesis^12^.

Myricetin is a widely distributed flavonol compound found in many plants, including tea, berries, fruits, vegetables, and medicinal herbs. A growing body of literature provided evidence that myricetin can prevent or decelerate the progression of various diseases, including cancer^18^, metabolic disease^19^, neurodegenerative diseases^20^, skin disease^21^ and inflammatory disorders^22^, as well as extend the lifespan of *Caenorhabditis elegans* ^23, 24^. Corresponding to its pleiotropic roles in improving health and preventing or treating chronic diseases, myricetin has been shown to target diverse cellular pathways under varying conditions^25^, revealing a mechanistic complexity that warrants further investigation.

In this study we investigated the function and mechanism of myricetin in energy homeostasis in metazoans. Studies in mice showed that myricetin potently induced mitochondrial activity, increased muscle strength, and improved cold resistance tolerance. These effects correlated with broad gene expression reprograming by myricetin in metabolically active tissues and required SIRT1-dependent PGC-1α deacetylation. Importantly, using *C. elegans* as an aging model, we showed that the anti-aging and motility-enhancing efficacies of myricetin were attenuated in SIR-2.1/SIRT1 mutant, yet remained largely intact in DAF-16/FOXO and AAK-2/AMPK mutant animals. Therefore, myricetin is a key regulator of mitochondrial function and energy homeostasis, via a mechanistic pathway requiring PGC-1α deacetylation by SIRT1.

## Results

### Myricetin improved the aerobic capacity in muscle

**Figure 1:**
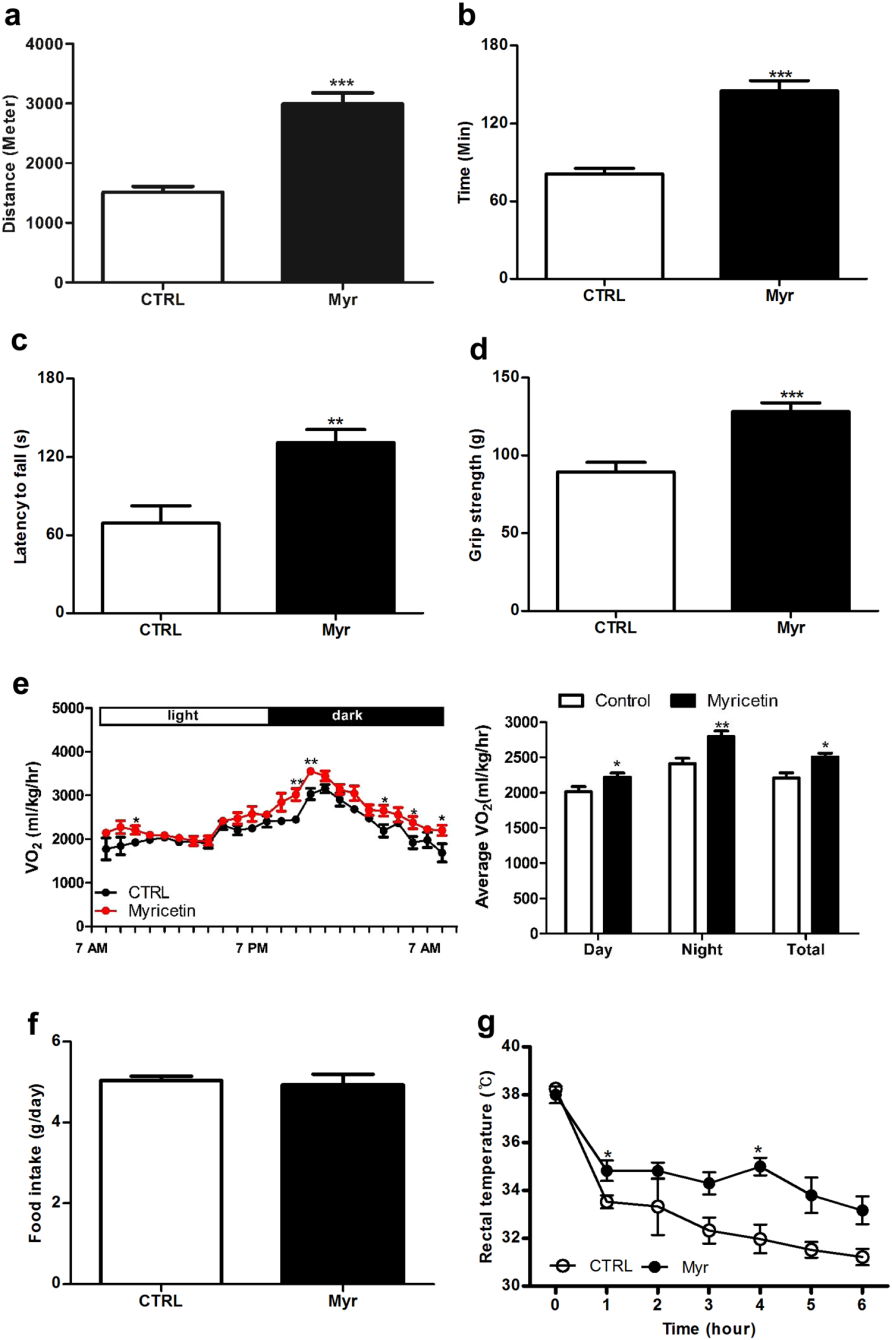
Myricetin improved motor function and energy expenditure (EE). (**a**,**b**) The effect of myricetin on endurance, as measured by a treadmill exercise test. The average run-until-exhaustion distance (**a**) and time (**b**) are presented for animals treated with PBS or myricetin at 50 mg/kg (n = 10 animals/group). (**c**,**d**) Behavior tests to evaluate mouse sensorimotor function using a rotarod (**c**), and grip strength test (**d**) (n = 10 animals/group). (**e**) EE as measured by changes in O_2_ consumption in indirect calorimetry over **24** hr. Average VO_2_ during light and dark cycles in mice administered myricetin or not are shown in the graph on the right (n = 6~8 animals/group). (**f**) Myricetin treated mice showed no effect on food intake of mice. (**g**) The internal body temperature during a cold tolerance test (4 °C for 6 hr). n = 4 animals/group. Unpaired Student’s t-tests, * = P < 0.05, ** = P < 0.005, *** = P < 0.0005. Values represent means ± SEM.

Previous studies have shown that decreased aerobic capacity and mitochondrial oxidative phosphorylation are associated with reduced longevity^12^. Myricetin is known to extend lifespan in *C*. *elegans*, yet it is unknown whether it impacts endurance and mitochondrial function. To address this question, especially in mammals, we first investigated physiological and behavioral effects of myricetin in mice. Male ICR mice were administered with myricetin at 50 mg/kg/day for 3 weeks, with a group of untreated mice as controls. Treadmill endurance test revealed that mice treated with myricetin displayed twice greater run-to-exhaustion distance and time compared with controls (Fig. 1a,b). In contrast, hot-plate pain test showed no difference in pain sensitivity between myricetin-treated and nontreated control mice (Figure S1e), excluding the possibility that enhanced endurance capacity might be due to altered pain sensitivity. Interestingly, relative to nontreated mice, the myricetin-treated mice displayed increased muscle strength (Fig. 1d) and markedly improved motor coordination in the accelerating rotarod test (Fig. 1c). These results, together with the data from the treadmill exercise test, suggest that myricetin improves motor function.

These beneficial effects of myricetin on motor function were not due to decreased body weight and food intake, as the amount (kcal) of food consumed per mouse over a 24 hr period and body weight were unchanged (Figure S1a). Myricetin, at the dose given, did not induce hepatic and other overt toxicity, since the liver and kidney histo-morphology were normal and the serum levels of alanine aminotransferase, aspartate aminotransferase and various markers (Table S1) were unchanged (Figure S1d). In addition, coat maintenance, stool composition, and water intake (data not shown) were unaffected, indicating that myricetin was well tolerated by the animals.

### Myricetin enhanced mitochondrial density in skeletal muscle and BAT

**Figure 2:**
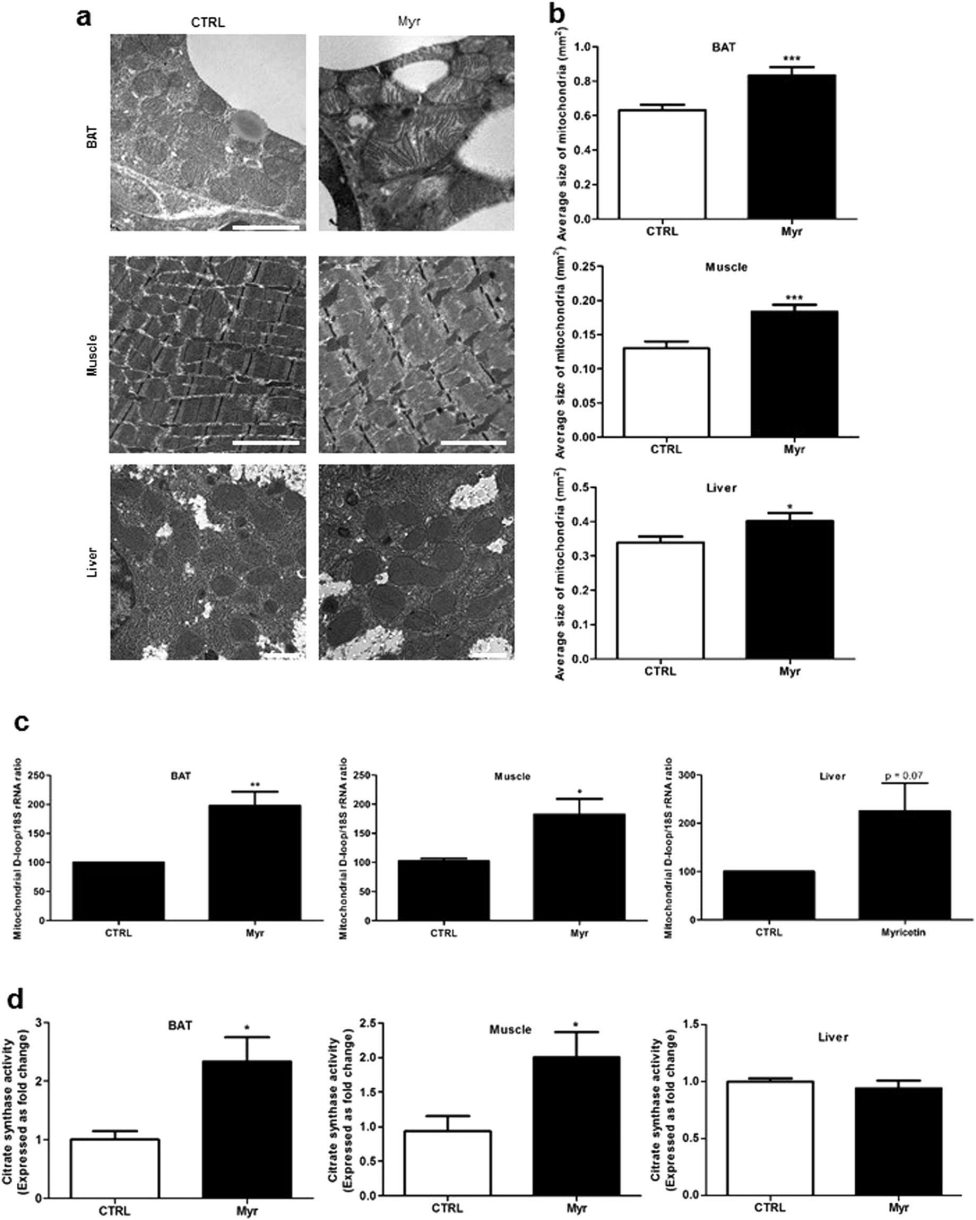
Myricetin increased mitochondrial activity in BAT and skeletal muscle. (**a**) Representative electron microscopy images of liver, BAT and skeletal muscle tissues from control and myricetin-treated mice. Scale bar: 2 μm. (**b**) Quantification of mitochondria area of the indicated tissue. Mitochondria area was measured in liver (91–117 individual mitochondria), BAT (94–134), and skeletal muscle (117–173). N = 3–4 animals/group. (**c**) D-loop regions of mitochondrial DNA (mtDNA) isolated from gastrocnemius muscle, BAT, and liver (n = 4 animals/group). (**d**) Activity of the citrate synthase, as measured with homogenates of BAT, liver, and gastrocnemius fibers isolated from myricetin-treated and nontreated mice (n = 3~6 animals/group). Unpaired Student’s t-tests, *=P < 0.05, **=P < 0.005, ***=P < 0.0005. Values represent means ± SEM.

Certain natural polyphenolic compounds, such as resveratrol, have shown endurance enhancing activities by promoting mitochondrial density and energy expenditure^12^. Next, we directly investigated whether the myricetin-mediated increase in muscle endurance enhancing activities were a result of metabolic enhancement, specifically elevated mitochondrial respiration. We first conducted indirect calorimetry to examine the effect of myricetin on energy expenditure (EE) in mice. Basal EE, as measured by oxygen (O_2_) consumption, was significantly increased in myricetin-treated mice (Fig. 1e), but their ambulatory locomotor activity and food intake were not changed (Figs S1c and 1f). To assess the effect of myricetin on adaptive thermogenesis, we performed a cold tolerance test. Myricetin-treated mice were found to better maintain body temperature compared with control mice, indicating its beneficial role in adaptive thermogenesis (Fig. 1g). In the mouse, the major site of heat production is the brown adipose tissue (BAT). Interestingly, morphometric analysis of BAT mitochondria, by electron microscopy, revealed clearly larger mitochondrial structures due to enriched cristae in myricetin-treated mice as compared to those in nontreated animals (Fig. 2a,b). In adult human, however, skeletal muscle is the predominant tissue providing the majority of mitochondrial capacity for EE. Mitochondria are dynamic organelles that can change in number and morphology within a cell and morphological dynamics of mitochondria are linked to regulation of many specific cell functions. The mitochondria of healthy cells exhibit elongated and tubular morphology, so we identified and quantified mitochondria morphology in each image, and the percent of elongated mitochondria was calculated. Myricetin-treated mice had a higher percentage of elongated mitochondria in BAT and liver compared with the nontreated mice (Figure S2). In skeletal muscle, myricetin-associated mitochondrial expansion was evidenced by increased mitochondrial size (Fig. 2a,b) and D-loop content (Fig. 2c). Similar changes were observed in C2C12 myotubes treated with myricetin (Figure S3a). We also observed increases in citrate synthase activity in muscle and BAT homogenates, further supporting that myricetin enhanced mitochondrial activity and consequently improved endurance and adaptive thermogenesis in mice (Fig. 2d). To extend the results on BAT and muscle, we next examined the effect on liver, another important mitochondria-rich metabolic organ. Measurement of mitochondrial mass by electron microscopy showed that myricetin increased mitochondrial area in treated mice relative to controls (Fig. 2a,b), as evidenced by both mitochondrial size increase (Fig. 2a,b) and D-loop content (Fig. 2c). However, citrate synthase activity in liver was unaltered by myricetin (Fig. 2d), indicating tissue-dependent variable effects of myricetin.

### Myricetin reprogramed gene expression in metabolically active tissues

**Figure 3:**
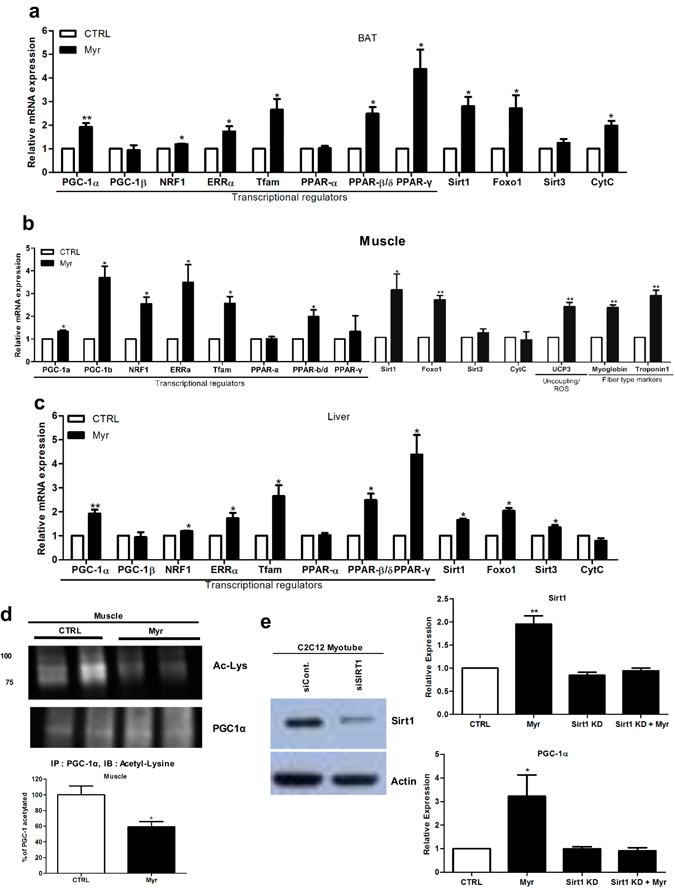
Myricetin increased gene expression important for mitochondrial function and biogenesis in mice. (**a**–**c**) Relative mRNA expression levels of key genes related to mitochondrial function as measured by RT-qPCR in the BAT (**a**), gastrocnemius muscle (**b**), and live (**c**) from PBS or myricetin-treated mice (n = 3~4 animals/group). Data represent mRNA levels relative to β-actin and are shown as means ± SEM. (**d**) Representative western blots and quantification showing the relative amount of acetylated versus total PGC-1 protein in gastrocnemius muscle. PGC-1α was immunoprecipitated (IP) from nuclear extracts and then immunoblotted with either an anti-acetylated lysine antibody to determine the extent of PGC-1α acetylation (Ac-Lys) or an anti-PGC-1α antibody to determine the total amount of PGC-1α. N = 4 animals/group. (**e**) C2C12 myotubes were transfected with a Sirt1 siRNA or a control siRNA. Following 24 hr incubation with DMSO or myricetin (100 μM), cells were harvested for protein and RNA extraction. A representative western blot showing SIRT1 protein expression in these cells is shown. Actin was used as a loading control. The mRNA expression levels of Sirt1 and PGC-1α were determined by RT-qPCR. Values represent the mRNA levels relative to β-actin, Unpaired Student’s t-tests, *=P < 0.05, **=P < 0.005, ***=P < 0.0005. Values represent means ± SEM.

To understand the molecular basis for the myricetin-mediated mitochondrial enhancement, we performed quantitative RT-PCR to examine expressions of genes important for mitochondrial biogenesis and functions in muscle, BAT and liver. We first measured the expression of PGC-1α and several other genes of its targets, as well as Sirt1^12^. Sirt1 and PGC-1α mRNA expression was significantly induced in these tissues from myricetin-treated mice (Fig. 3a–c). The gene encoding nuclear respiratory factor-1 (NRF-1), indispensable for the expression of key mitochondrial encoded genes^26–28^, was also markedly increased by myricetin. Several other PGC-1α targets were also activated^29^, including genes involved in fiber-type markers (MB: myoglobin) as well as PPARβ/δ genes. Of note, it has been shown that PPARβ/δ agonists increase oxidative myofibers and running endurance in adult mice^30^. Similar gene induction results were observed in C2C12 myotubes (Figure S3b).

As predicted from the electron microscopy and cold tolerance test results, we noted a significant increase in BAT gene expression related to energy homeostasis (Fig. 3a). In particular, PGC-1α, PPARα, and UCP-1 mRNA levels were all induced by myricetin. PPARα activates genes important for β-oxidation of fatty acids^31^, and UCP-1 is mainly responsible for heat production in BAT via the uncoupling of respiration from ATP synthesis^32^. In liver, myricetin also induced increased expression of PGC-1α, PPARβ/δ, and PPARγ along with Sirt1. However, myricetin-induced gene expression showed tissue specificity in those tested, suggesting that myricetin differentially regulates signaling pathways involved in energy homeostasis in distinct metabolic tissues.

### Myricetin induced PGC-1α activity through SIRT1

Apart from gene regulation (Fig. 3a–c), PGC-1α can also be modulated at the posttranslational level by protein modification with acetylation, which significantly impacts its activity^14^. Therefore, we compared PGC-1α acetylation in skeletal muscle between the treatment groups (Fig. 3d). In gastrocnemius muscle, we observed that the ratio of acetylated vs. total nuclear PGC-1α protein was significantly decreased in myricetin-treated mice, suggesting that PGC-1α activity was also increased (Fig. 3d). We found that deacetylase activity of SIRT was associated with an increase in the NAD^+^ levels in muscle from myricetin administered mice compared to vehicle group (Figure S4a). This is in line with a slight increase in NADH levels (Figure S4b) and an increase in the NAD^+^∶NADH ratio (Figure S4c). To determine whether the effect of myricetin on mitochondrial function was mediated by SIRT1^12^, we transfected C2C12 myotubes with a specific short interfering RNA (siRNA) directed against Sirt1 or a control siRNA. Sirt1 siRNA was found to effectively reduce endogenous SIRT1 levels (Fig. 3e, left panel). Interestingly the SIRT1 knockdown largely blocked the myricetin-induced PGC-1α expression (Fig. 3e, right panels). These results indicate bimodal roles of myricetin in PGC-1α induction, namely both gene activation^33^ and post-translational deacetylation by SIRT1.

### Myricetin improved locomotion and delays aging through sir-2.1/Sirt1 in *C. elegans*

**Figure 4:**
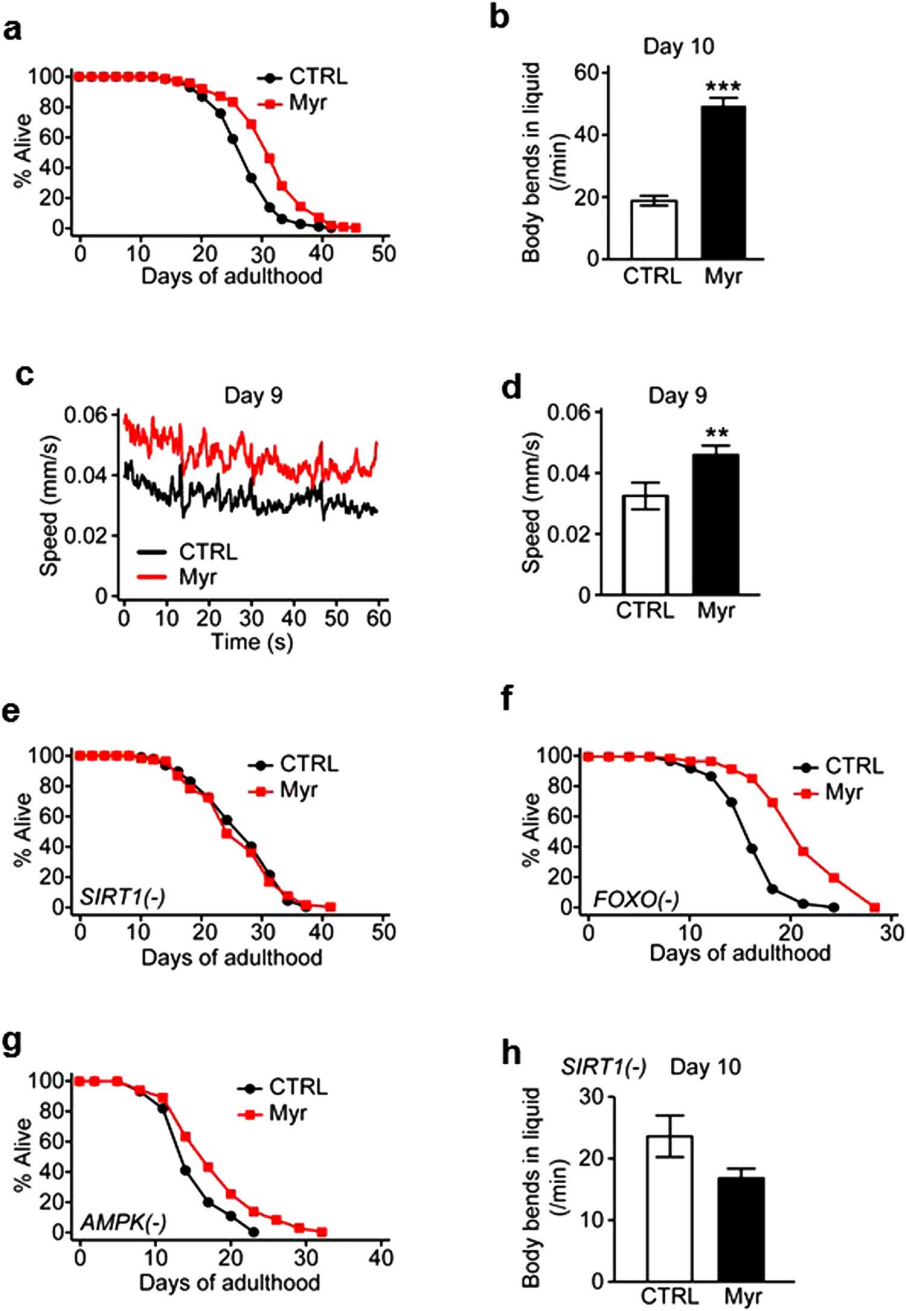
Myricetin improved locomotion and delayed aging in *C. elegans* via SIRT1. (**a**) Myricetin increased the lifespan of wild-type *C. elegans*. (**b**) Myricetin treatments increased the body bending rates of old (day 10 adult) wild-type *C. elegans*. (**c**) Enhanced motility of myricetin-treated worms measured by Multi-Worm Tracker at day 9 of adulthood. (**d**) Average speed of the worms measured for 1 min shown in panel B (n ≥ 11). (**e**) SIRT1/*sir-2.1(ok434)* [SIRT1(−)] mutations suppressed the lifespan-extending effect of myricetin. (**f**,**g**) Myricetin extended the lifespan of FOXO/*daf-16(mu86)* [FOXO(−)] mutants (**f**) and AMPK/*aak-2(ok524)* [AMPK(−)] mutants (**g**). (**h**) Myricetin treatments did not increase the body bending rates of old (day 10 adult) SIRT1 mutants (n = 15). Error bars represent standard error of the mean (SEM) (two-tailed Student’s t-tests, **p < 0.01). See Supplemental Table S2 for additional repeats and statistical analysis.

We then examined the role of myricetin in organismal aging by using the roundworm *C. elegans*, an excellent model animal for aging research. Consistent with previous reports^23, 24^, we found that myricetin treatment significantly increased the lifespan of wild-type *C. elegans* (Fig. 4a). In addition, myricetin also markedly enhanced the mobility of aged worms (Fig. 4b–d), compared with a diminished effect in young worms (Figure S5a–S5c). These results suggest that myricetin delays aging in *C. elegans*. Next, we asked which genetic factors mediate the anti-aging effect of myricetin. SIR-2.1/SIRT1, DAF-16/FOXO and AAK-2/AMPK are among key anti-aging longevity factors that are conserved in many species^34^. We therefore measured the lifespan of mutant worms that are defective in each of these three genes with or without myricetin treatment. Remarkably, myricetin did not increase the lifespan of *SIRT1* mutant (Fig. 4e), while significantly lengthening that of *FOXO* or *AMPK* mutants (Fig. 4f,g). In accordance, myricetin failed to enhance the locomotion capacity of aged *SIRT1* mutants (Figs 4h and S5d). These data suggest that myricetin treatment delays organismal aging in a SIRT1-dependent manner.

## Discussion

In summary, our current study reveals a robust effect of the natural flavonol myricetin in mouse energy metabolism. Using physiological and behavioral approaches, we showed that myricetin improved mouse energy expenditure and mitochondrial function in metabolically active organs including skeletal muscle and BAT. In contrast, no effect on mitochondrial function was observed in liver (Fig. 2d), indicating a certain tissue specificity in myricetin’s effects. Molecular analyses revealed a bimodal effect of myricetin on PGC-1α expression induction and post-translational deacetylation via SIRT1, both serving to promote mitochondrial function and biogenesis. Using *C. elegans* as an aging model, we showed that the anti-aging effect of myricetin is critically dependent on SIRT1, illustrating a mechanistic pathway involving SIRT1 and PGC-1α that mediates the beneficial effects of myricetin on mitochondrial function and physiological well-being.

The PGC-1α expression was found to decrease in skeletal muscles from mice lacking the sirtuin family deacetylase SIRT^33^. Conversely, SIRT1 overexpression or activation in differentiated C2C12 myotubes increased PGC-1α mRNA expression^33^. On the other hand, myricetin^35^, also induces PGC-1α activity by facilitating SIRT1-mediated deacetylation. Changes in NAD^+^ are generally translated into altered SIRT1 activity^9^. The higher NAD^+^ levels in myricetin administered mice were indeed reflected in hypoacetylation of the SIRT1 substrate PGC-1α, indicative of increased SIRT1 activity (Figs 3d and 4a). The effects of myricetin on PGC-1α gene expression were dependent on the presence of the SIRT1 and were lost when SIRT1 expression was downregulated in C2C12 myotubes by siRNA. These observations together support dual roles of myricetin and SIRT1 in promoting PGC-1α expression and activity.

Impaired mitochondrial function is also associated with rapid onset of symptoms commonly seen in the elderly, such as type 2 diabetes, neurodegeneration, and muscle loss^36–38^ and treatments that enhance mitochondrial function can delay the progression of these age-related diseases^12, 39, 40^. In addition to its locomotion function, skeletal muscle is also a pivotal tissue responsible for insulin-stimulated glucose uptake and hence energy homeostasis, and reduced mitochondrial function in muscle has been shown to play an essential role in the development of insulin resistance with obesity^41^. Together, activation of pathways upstream of mitochondrial biogenesis, such as myricetin-dependent PGC-1α activation, may show efficacy in treating or delaying these debilitating diseases.

Previous studies examined the role of FOXO in the myricetin-induced longevity in *C. elegans*, but other longevity factors were not experimentally tested^23, 24^. In this study, we investigated several key regulators of aging and metabolism such as SIRT1, FOXO, and AMPK^42^. Consistent with our mouse functional data, the worm SIRT1 homolog appeared to mediate the longevity effect of myricetin in *C. elegans*. SIRT1 is also known to mediate the lifespan extension by CR in *C. elegans* ^43^. As CR is known to promote mitochondrial activity^44^, the mitochondria-enhancing mechanism by which myricetin extends lifespan and healthspan is likely to be conserved from nematodes to mammals. However, *C. elegans* does not express a PGC-1α homolog^29^, suggesting a novel factor mediating the lifespan-extending effects of myricetin downstream of SIRT1 in *C. elegans*. Our data showing that myricetin increases the lifespan of *FOXO(-)* is consistent with a previous report^23^, but not with another study reporting that FOXO is required for the myricetin-induced longevity^24^. In our experimental conditions, we used ethanol mixed with detergent (Tween 80) as a solvent control as previously described^23^, whereas Buchter *et al*. used DMSO as a solvent^24^. In light of the demonstrated effect of DMSO on the lifespan of *C. elegans* ^45^, different experimental conditions such as solvents used may have resulted in, or contributed to, the functional differences. Together, our *C. elegans* data suggest that myricetin increased organismal lifespan and healthspan in a SIRT1-dependent manner. Further research will examine whether myricetin feeding can delay aging in mammals, which may ultimately accelerate the application of myricetin for general health and longevity.

## Materials and Methods

### Cell Culture and Molecular Biology Studies

C2C12 mouse myoblasts were maintained in DMEM (4.5 g glucose/L) supplemented with 10% fetal bovine serum, 1% Anti-Anti and cells were maintained at lower than 80% confluence. Differentiation to myotubes was induced over 5 days by replacing the medium of confluent cultures with DMEM containing 2% horse serum. Before myricetin (Sigma) treatment, cells were washed with PBS and incubated with DMEM containing 0.5% FBS treated for 24 hr with myricetin (10 or 30 or 100 μM) or DMSO. Methods for molecular assays are provided in the Supplemental Experimental Procedures.

### *In Vivo* Analysis

Seven-week-old male ICR mice from Koatech Co. Ltd (Gyeonggi-do, Korea). All animal experiments were approved by the Ethics Review Committee of the Pohang center for the evaluation of biomaterials, Republic of Korea. Mice were housed in specific pathogen-free conditions with a 12 hr light-dark cycle and had free access to water and food. Body weight and caloric intake were monitored throughout the experiments. The mice were housed together and food intake was calculated of animals in the cage. 50 mg/kg/day myricetin or PBS, as the vehicle control, was orally administered daily for 3 weeks. The mice were sacrificed at the following ages: 3 months by cervical vertebral dislocation, and liver (corresponding to left lobe), kidney, interscapular BAT, and gastrocnemius muscle were collected. The protocols used to assess behavioral, and metabolic phenotypes included the following: energy expenditure by indirect calorimetry (12 hr, food and water) and 4 °C cold test(6 hr); circadian activity by metabolic cage monitoring (24 hr); locomoter function by rotarod, grip strength test; and endurance test by variable speed belt treadmill and incremental speed protocol (range from 18 cm/s to 41 cm/s on habituated 2 hr fasted mice). Details are in the Supplemental Experimental Procedures.

### Metabolic phenotypes

Metabolic analysis: To measure the food intake, physical activity, and metabolic rate, the mice were housed individually in a TSE indirect calorimetry system (Lab Master, TSE systems) for 24 hr with 12 h light/dark cycles at 22 ± 2 °C.

Thermogenesis test: The adaptive thermogenic response was measured in a cold tolerance test, during which the animals were individually housed at 4 °C for 6 hours.

### Electron Microscopy

Liver (corresponding to left lobe), interscapular BAT, and gastrocnemius muscle were removed from mice and immediately put in 4% paraformaldehyde containing sodium cacodylate buffer (50 mM sodium cacodylate, pH 7.2) and kept at 4 °C. The tissues were then washed three times with sodium cacodylate buffer followed by postfixation with 1% osmium tetroxide in 50 nM sodium cacodylate buffer for 2 h at 4 °C. The samples were treated with 0.5% uranyl acetate at 4 °C for 30 min, followed by dehydration with a graded series of ethanol. Dehydrated samples were infiltrated with a graded series of propylene oxide and resin mix before to be embedded in 100% Spurr’s resin which was polymerized at 70 °C for 24 hr. Ultrathin sections were cut at 70 nm with an ultramicrotome (MT-X, RMC, Tucson, AZ, USA) and contrasted with 2% uranyl acetate and Reynolds’ lead citrate. Samples were examined with JEM-1011 electron microscope (JEOL, Tokyo, Japan). Our mitochondrial area measurements were largely similar to those described previously with minor modifications^12^. Briefly, for analyzing individual mitochondrial area, electron micrographs were digitized and the area of each clearly distinguishable mitochondria was analyzed using ImageJ. For each animal, regions containing numerous mitochondria were analyzed in BAT, muscle or liver, for which at least 5 micrographs were captured, from different area of one grid. Mitochondria with length:width ratio>1.5 were considered as elongated mitochondria. Mitochondria containing these morphologies were counted and expressed as a percent of the total mitochondria. Mitochondrial areas and morphology of at least 90 mitochondrial profiles from each group were measured.

### Histology

In preparation for hematoxylin and eosin (HE) staining, the tissues were fixed in 10% formalin and embedded in paraffin. Sections of 3-μm thickness were affixed to slides, deparaffinized, dehydrated, and then stained with HE. Stained liver and kidney sections were observed under light microscopy (BX 50, Olympus, JAPAN). Each sample was observed at 400X magnification. The liver and kidney tissues from one mouse from the control, and 50 mg/kg myricetin treated groups were analyzed.

### Serum analysis

Serum was collected by cardiac puncture, stored for 20 minutes at room temperature for coagulation and then separated by centrifugation at 2,000 × g for 40 minutes. The serum was stored at −70 °C until analysis. The levels of total protein, albumin, creatinine, triglycerides, total cholesterol, high‐density lipoprotein (HDL)‐cholesterol, low‐density lipoprotein (LDL)‐cholesterol, uric acid, glucose, lactate dehydrogenase, alanine transaminase (ALT), aspartate transaminase (AST) and creatinine kinase in serum were measured by using an auto chemistry Equalizer (BS-390, Mindray Bio-medical Electronics Co., Ltd., China).

### *C. elegans* study

Methods for *C. elegans study* are provided in the Supplemental Experimental Procedures.

### Statistical analysis

Statistical analyses were performed with Prism software (GraphPad Prism 5, USA). We used student’s t test (two-tailed) for mice behavior, metabolic phenotypes, *ex-vivo* analysis, siRNA experiment and *C. elegans* study. For the multi dose of myricetin *in vitro* data, we applied one-way ANOVA followed by Tukey’s post hoc test. A *P* value < 0.05 was considered statistically significant.

### Ethics Statement

Approval of the study protocol was obtained from the Advanced bio convergence center and Technology Institutional Animal Care and Use Committee (approval number: POCEB-011). All animal experiments were carried out according to the provisions of the Animal Welfare Act, PHS Animal Welfare Policy, and the principles of the NIH Guide for the Care and Use of Laboratory Animals. All mouse lines were maintained at the Advanced Bio Convergence Center animal facility under institutional guidelines.

## Electronic supplementary material

Supplementary informaiton
