# Supplementary material for: Myricetin improves endurance capacity and mitochondrial density by activating SIRT1 and PGC-1α
Source: Sci Rep. 2017 Jul 24;7:6237. doi: 10.1038/s41598-017-05303-2 (PMC5524912; doi:10.1038/s41598-017-05303-2)
Supplement: Supplementary file 1 — Supplementary informaiton [file 41598_2017_5303_MOESM1_ESM.doc]

***Supplemental information for***

**Myricetin improves endurance capacity and mitochondrial density by activating SIRT1 and PGC-1α**

**Hoe-Yune Jung^1, 5, 6^, Dongyeop Lee^2^, Hye Guk Ryu^2^, Bo-Hwa Choi^6^, Younghoon Go^7, 8, 10^, Namgyu Lee^2^, Dohyun Lee^2^, Heehwa G. Son^2^, Jongsu Jeon^5^, Seong-Hoon Kim^2^, Jong Hyuk Yoon^2^, Seon-Min Park^6, 11^, Seung-Jae V. Lee^2, 3, 4^, In-Kyu Lee^7, 8^, Kwan Yong Choi^1, 2^, Sung Ho Ryu^1, 2^, Kazunari Nohara^9^, Seung-Hee Yoo^9^, Zheng Chen^9^ and Kyong-Tai Kim^1, 2, 3^, ***

^1^Department of Integrative Biosciences & Biotechnology, Pohang University of Science and Technology(POSTECH), 77 Cheongam-Ro, Pohang, Gyeongbuk, 37673 Republic of Korea

^2^Department of Life Science**s**, Pohang University of Science and Technology(POSTECH), 77 Cheongam-Ro, Pohang, Gyeongbuk, 37673 Republic of Korea

^3^Information Technology Convergence Engineering, Pohang University of Science and Technology(POSTECH), 77 Cheongam-Ro, Pohang, Gyeongbuk, 37673 Republic of Korea

^4^School of Interdisciplinary Bioscience and Bioengineering, Pohang University of Science and Technology(POSTECH), 77 Cheongam-Ro, Pohang, Gyeongbuk, 37673 Republic of Korea

^5^R&D Center, NovMetaPharma Co., Ltd., 394 Jigok-Ro, Pohang, Gyeongbuk, 37668 Republic of Korea

^6^Advanced Bio Convergence Center, Pohang Technopark, 394 Jigok-Ro, Pohang, Gyeongbuk, 37668 Republic of Korea

^7^Department of Internal Medicine, Kyungpook National University School of Medicine, Daegu, 41944 Republic of Korea

^8^Leading-Edge Research Center for Drug Discovery and Development for Diabetes and Metabolic Disease, Kyungpook National University Hospital, Daegu, 41404 Republic of Korea

^9^Department of Biochemistry and Molecular Biology, The University of Texas Health Science Center at Houston, 6431 Fannin St., Houston TX 77030 USA

^10^Korean Medicine (KM) Application Center, Korea Institute of Oriental Medicine (KIOM), Daegu, 41062 Republic of Korea

^11^Department of Veterinary Medicine, Kyungpook, National University, Daegu, 41566 Republic of Korea

*Corresponding author information:

Kyong-Tai Kim, Ph.D., Professor

Division of Integrative Biosciences & Biotechnology, POSTECH,

Hyoja Dong, Pohang, Gyeongbuk, Korea, 790-784

E-mail : ktk@postech.ac.kr

Tel: +82-54-279-2297

**
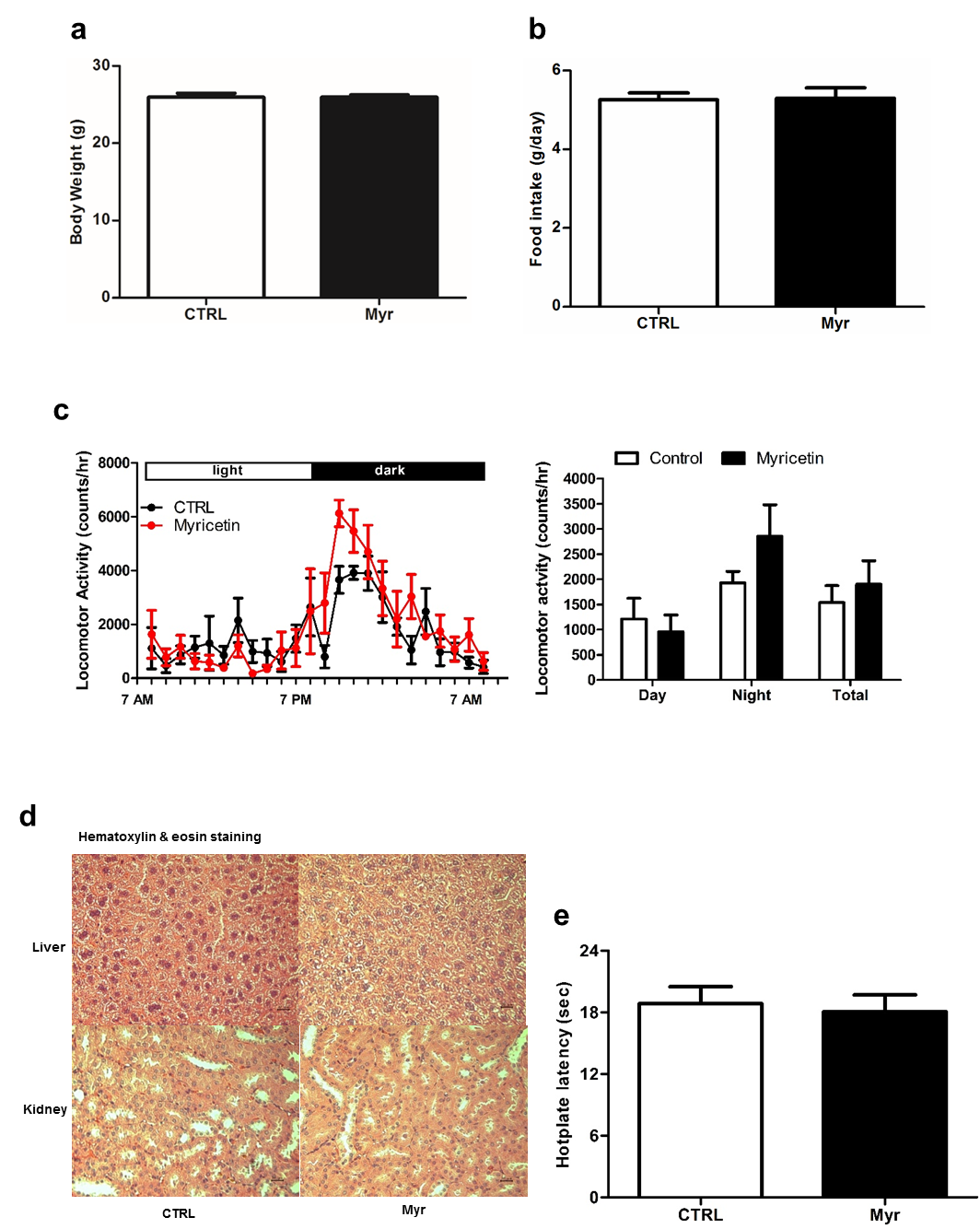
**

**Figure S1 related to Figure 1. No change in metabolic features and pain sensitivity was observed between control and myricetin treated groups.**

(**a** - **f**) Myricetin showed no effect on body weight (**a**), food intake (**b**), locomotor activity (**c**), biochemical parameters (**d**), histological evaluation of liver and kidney (**e**) and pain sensitivity (**f**) of mice*.* Error bars represent standard error of the mean (SEM)

**
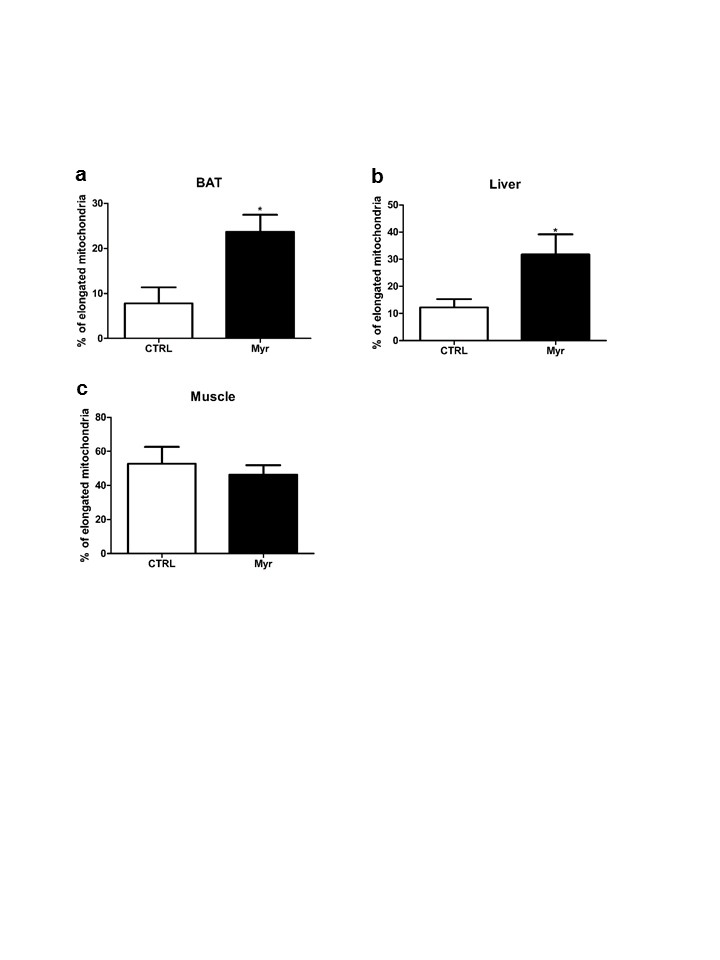
**

**Figure S2 related to Figure 2. Myricetin increases the proportion of elongated mitochondria in BAT and Liver.**

(**a-c**) Percentages of cells with elongated mitochondrial morphology. At least 90 mitochondria in each group were counted (mean ± s.e.m., Unpaired Student’s t-tests, *P<0.05, **P<0.01).


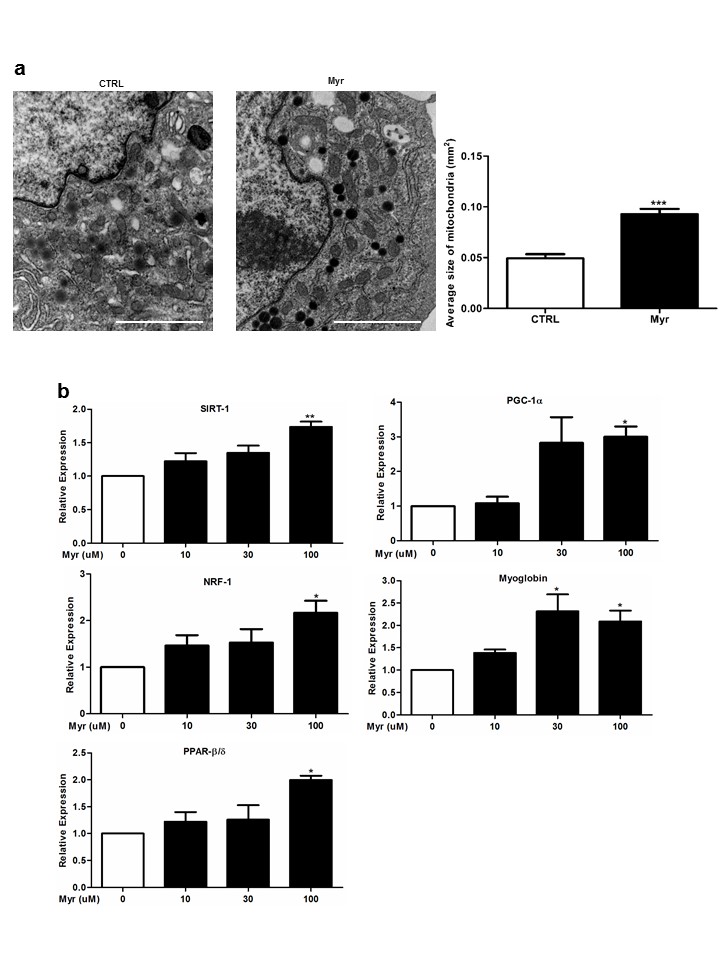


**Figure S3 related to Figure 2-3. Myricetin increased mitochondrial activity and mRNA expression of genes related to mitochondrial biogenesis in C2C12 myotubes.**

(**a**) Transmission electron microscopy (TEM) images from C2C12 myotubes treated with DMSO or myricetin. Scale bar: 2㎛. Mitochondrial morphology quantification in EM was measured with ImageJ, represented as mitochondria area, using 7 different images (magnification of 20K, 25K and 40K) at each condition (n=100 to 172 mitochondria/group). (**b**) Relative mRNA expression levels of essential genes related to mitochondrial function as measured by RT-qPCR in C2C12 myotubes (n=3). Data represent mRNA levels relative to β-actin and are given as means ± SEM. TEM images were analyzed by unpaired Student’s t-tests and mRNA expression was applied one-way ANOVA followed by Tukey's post hoc test, * = P < 0.05, ** = P < 0.005, *** = P < 0.0005.


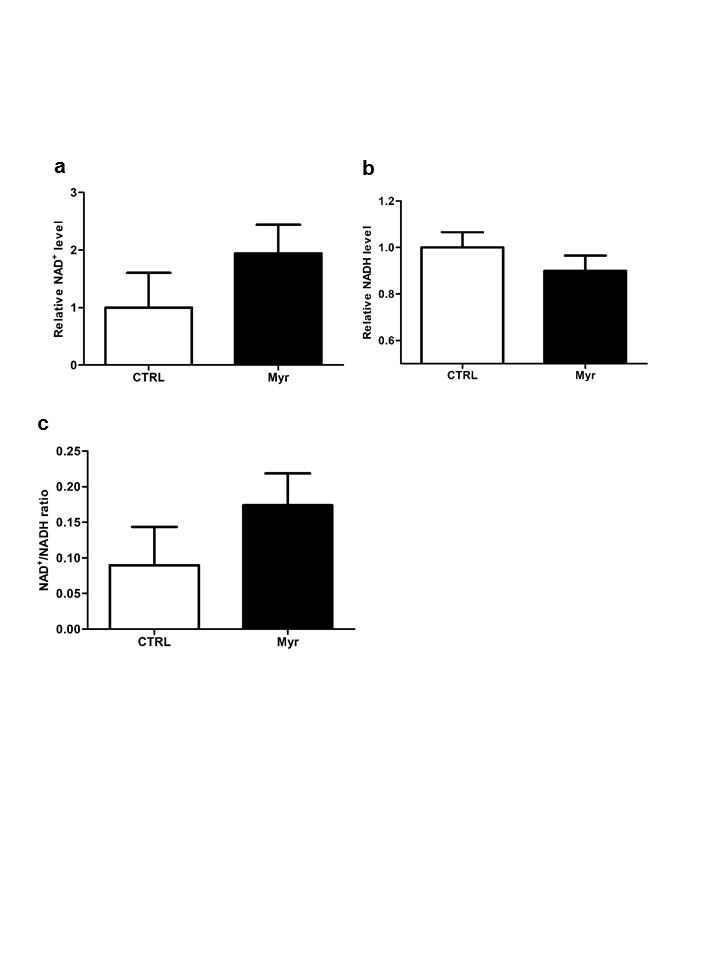


**Figure S4 related to Figure 3. Myricetin treated mice increased NAD^+^ levels important for deacetylase activity of SIRT1**

(**a**) Myricetin increase NAD^+^ level, NAD^+^∶NADH ratio (**c**) and decrease in NADH levels (**b**). The values denote means ± SEMs.


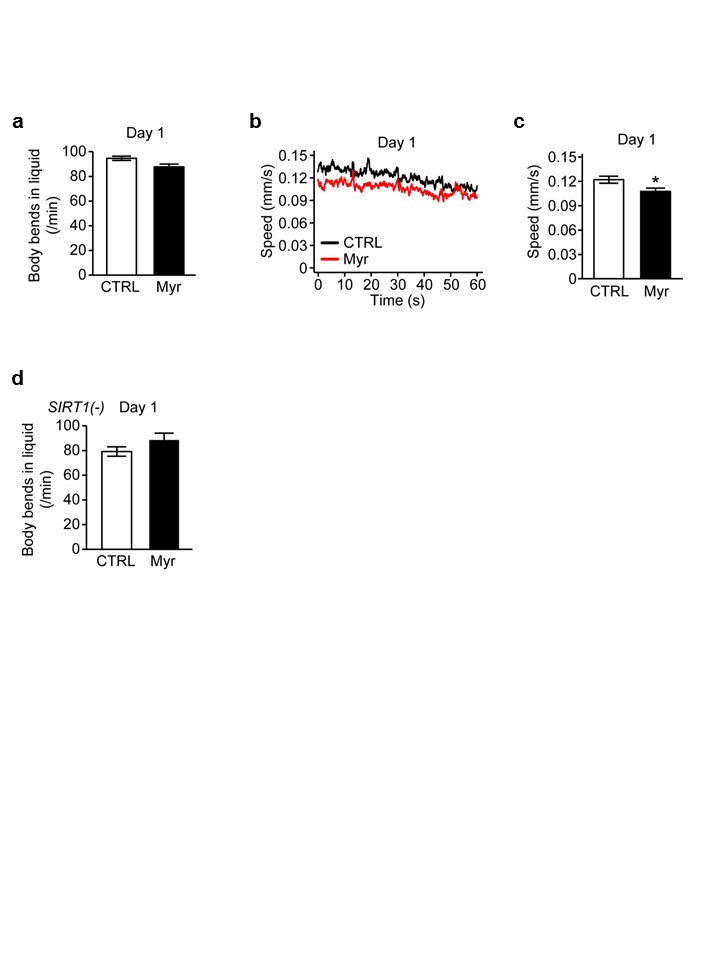


**Figure S5 related Figure 4. Myricetin showed little or no effect on locomotion of young *C. elegans*.**

(**a**) Myricetin had no effect on the body bending of young (day 1 adult) wild-type *C. elegans.* (**b**) Myricetin treatment slightly decreased the motility of *C. elegans* at day 1 of adulthood. (**c**) Average speed of the animals for 1 min shown in panel **A** (n≥11). (**d**) Myricetin treatments did not significantly change the body bending of young (day 1 adult) *SIRT1*/*sir-2.1(ok434)* [*SIRT1(-)*] mutants. Error bars represent standard error of the mean (SEM) (two-tailed Student’s t-tests, *p<0.05)

**Behavioral tests.**

*Endurance tests:* Mouse endurance was measured on a treadmill (Panlab, Harvard Apparatus, Spain) enclosed in a plexiglass chamber that was outfitted with a shock grid at the rear of the belt to keep the animal running during the test. The shock grid delivered a 1mA shock, which was uncomfortable but would not physically harm or injure the animals. Animals were habituated to the test conditions before experimentation. In the test procedure, mice ran on a treadmill with a 5° incline at a speed of 27 cm/sec for 10 min. For the actual test, the speed was first set at 25 cm/sec with a 5° incline. The speed was then gradually increased from 25 to 41 cm/sec and was maintained at 41 cm/sec until exhaustion ^1^. The distance run and the number of shocks obtained over 5 min intervals were recorded and a mouse was considered exhausted and removed from the experiment when it received approximately 100 shocks in a period of 5 min.

*Accelerating rotarod test:* Motor coordination and balance were tested using an accelerating rotarod (Panlab, Harvard Apparatus, Spain). The rotarod test was performed by placing a mouse on a rotating drum and measuring the time each animal was able to maintain its balance walking on top of the rod. The speed of the bar was gradually increased from 4 to 40 rpm over a 5-min session. The mice were given four successive trials, and latencies to fall from the rotarod were recorded. The best trial (longest latency to fall) for each mouse was recorded.

*Grip strength test:* The grip test measured the maximal muscle strength (g) using an isometric dynamometer connected to a grid. A grip strength meter (Bioseb, France) was used to measure the grip strength of mice. Forelimb measurement was performed. Five measures of each were taken and means were calculated.

*Locomotor activity:* Locomotor activity was measured by using actimetry boxes (45 X 45 cm; Harvard Apparatus, Panlab, Spain) placed in a sound-proof cupboard. Movements were monitored via a grid of infrared beams and used as an index of locomotor activity (counts). Counts were integrated every hour and added to obtain total locomotor activity for a 24-h period. All data were collected with Acti-Track software (Harvard Apparatus). It measured as the total ambulatory locomotor activity.

*Hot plate test:* The surface of the hot plate was heated to 55 °C, as measured by both a built-in thermometer and an external surface thermometer. The mice placed on the surface of the heater and observed it until it responded to the thermal stimulus by a lick, hindpaw flick and/or jump; the latency of response of each mouse was recorded to the nearest millisecond.

**Citrate Synthase Activity.**

Citrate synthase assay was performed utilizing 10 μg of protein and a kit from Sigma (CS0720) as per the manufacturer's protocol. Briefly, Liver, BAT, and muscle tissues extracts were incubated with oxaloacetate and acetyl-CoA in a suitable buffer at 37 °C to generate CoA–SH, a thiol subsequently detected by a reaction with 5,5′-dithiobis (2-nitrobenzoic acid) to produce absorbance at 412 nm. Activity was normalized against equal amounts of protein.

**Preparation of nuclear and cytoplasmic extracts.**

Skeletal muscle tissue lysates were resuspended in hypotonic buffer (10mM HEPES, 10mM KCl, 1.5mM MgCl2, 1mM DTT, 0.2mM PMSF, 0.5% Nonidet P-40) and incubated at 4℃ for 30min. Samples were agitated every 10min and then centrifuged at 1800 x g for 4 min to collect the cytoplasmic fractions. To isolate nuclei, pellets were washed three times with and resuspended in nuclear extraction buffer (20mM HEPES, 450mM NaCl, 1.5mM MgCl2, 1mM DTT, 0.2mM PMSF) for 20min. Freeze-thawing was then repeated 5 times. The nuclear suspension was centrifuged at 16000 x g for 20 min, and the supernatants were recovered as the nuclear fractions.

**Immunoprecipitation and Immunoblotting.**

Nuclear extracts were resuspended in a reaction buffer (20mM Tris-HCl, pH7.5, 150mM NaCl, 0.5% Triton X-100, 1mM EDTA, 1mM PMSF). Equal amounts of protein were immunoprecipitated using anti-PGC-1 (Santa Cruz Biotechnology) and collected with Protein A/G-Sepharose beads (Santa Cruz Biotechnology) at 4℃ for 16 h. The immunoprecipitate was then washed 4 times in the cold reaction buffer described above. The bound proteins were resolved by SDS-PAGE and were transferred to nitrocellulose membranes. The membranes were blocked with 5% dry milk in TBS-T (20mM Tris-HCl, pH 7.5, 140mM NaCl, and 0.05% Tween-20) and subsequently incubated with the primary antibody Acetylated-Lysine (Cell signaling Technology). Specific reaction bands were detected using a goat anti-rabbit or goat anti-mouse IgG conjugated to horseradish peroxidase, and the immunoreactive bands were visualized with a SUPEX Western blotting detection kit (Neuronex, Korea).

**Assay of NAD^+^ and NADH**

Nicotinamide adenine dinucleotide oxidized form (NAD^+^) and nicotinamide adenine dinucleotide reduced form (NADH) concentration in the skeletal muscle of myricetin administered mice and vehicle group were determined using an NAD^+^/NADH Kit (K337-100; BioVision, Mountain View, CA, USA), as described by the manufacturer. Briefly, skeletal muscle tissues were homogenized and extracted with 1 ㎖of NADH/NAD extraction buffer; 0.2 ㎖of the extracted samples was heated at 60 °C for 30 min for NADH measurement. It was measured spectrophotometrically at 450 nm at room temperature.

**SiRNA.**

Lipofectamine 2000 (Invitrogen) was used to transfect siRNAs into cells. Small interfering RN**A** (siRNA) duplex targeting human SIRT1 and negative control siRNA were purchased from Bioneer (Korea). The target sequences of the siRNAs are followings. siSIRT1: 5-CUA AUC UAG ACC AAA GAA U-3, siCont.: 5-CCU ACG CCA CCA AUU UCG U-3.

**Analysis of mitochondrial DNA and biogenesis related factors by quantitative PCR (Q-PCR).**

D-loop is the initial region of mitochondrial DNA replication, so its copy number can indicate the level of mitochondrial DNA replication. Skeletal muscle tissue, BAT, liver and C2C12 myotubes were extracted with a QIAamp DNA Mini kit (Qiagen, Germany) according to the instructions and Q-PCR, using an iCycler iQ Real-Time Detection System (Bio-Rad, USA), was conducted with 18S rRNA primers for a nuclear target sequence and mitochondrial D-loop primers for a mitochondrial DNA target, respectively. The cycling conditions were as follows: 95 °C for 3 min, followed by 39 cycles at 95 °C for 10 sec, 58 °C for 10 sec and 72 °C for 30 sec. Each Q-PCR was performed in triplicate. The following primers were used: mitochondrial D-loop region forward: 5′-AAG GAC ATA TCT GTG TTA TCT GAC-3′ and reverse: 5′- TTC ACG GAG GAT GGT AGA TTA-3′; 18S rRNA forward: 5′-CAG TAA GTG CGG GTC ATA A-3′ and reverse: 5′-CCA TCC AAT CGG TAG TAG C-3′. The mouse 18S rRNA gene was served as the endogenous reference gene. The standard curve was drawn for relative quantification. The ratio of mitochondrial D-loop to 18S rRNA was then calculated. Final results are presented as a percentage of the control.

**RNA preparation and real-time PCR.**

Total RNA was prepared by an RNeasy Mini kit (QIAGEN) according to the manufacturer's instructions. RNA integrity was assessed by an automated microfluidics-based system (Bioanalyzer 2100, Agilent, Palo Alto, CA, USA). First-strand cDNA was synthesized with the iScript cDNA Synthesis Kit (Bio-Rad, Hercules, CA, USA), and real-time PCR was performed using an iCycler iQ Real-Time Detection System (Bio-Rad, USA). PCR reactions were carried out with iQ SYBR Green Supermix (Bio-Rad). Specific primer pairs (Genotech, Korea) are listed in supplemental table 3. β-actin was used as an internal control. Amplification of real-time PCR was performed according to the protocols of Jung *et al.*^2^ with modification. The reaction was carried out at 95 °C for 3 min and followed by 39 cycles of amplification (95 °C for 10 sec, 58 °C for 10 sec, 72 °C for 30 sec). A melt curve was produced to confirm a single gene-specific peak and detect primer/dimer formation by heating the samples from 65 to 95 °C in 0.5 °C increments with a dwell time at each temperature of 10 sec while continuously monitoring fluorescence. The mRNA levels of specific genes were normalized to those of β-actin.

***C. elegans* study.**

*C. elegans strains:* All *C. elegans* strains were maintained on solid nematode growth media (NGM) seeded with *E. coli* (OP50) at 20°C. Following strains were used in this study. N2 wild-type, CF2732 *sir-2.1(ok434) IV*, CF1042 *daf-16(mu86) I*, CF2725 *aak-2(ok524) X*.

*Preparation of myricetin-added plates:* Myricetin-treated plates were prepared as reported previously with some modifications ^3^. Myricetin was dissolved in ethanol:Tween 80 (92:8) as a stock solution (25 mM). The 100 ㎕ of stock solution was added onto *E. coli* OP50-seeded NGM media (5 ml) for 500 μM final concentration, and the same amount of ethanol:Tween 80 solution was added for solvent control.

*Lifespan assays:* Lifespan assays were performed as previously described ^4^. Three or four gravid adults were cultured on *E. coli* OP50-seeded NGM plates containing myricetin or solvent overnight to obtain synchronized progeny. When the progeny became young adults (day 1), the worms were transferred onto new myricetin-treated or control *E. coli* OP50-seeded NGM plates containing 50 μM 5-fluoro-2'-deoxyuridine (FUdR, Sigma, St Louis, MO, USA) to prevent progeny production. Worms that did not respond to gentle touching with a platinum wire were scored as dead. Worms that crawled off, ruptured or had internally hatched progeny were censored but included in subsequent statistical analysis. Lifespan data were analyzed by using OASIS (online application of survival analysis, http://sbi.postech.ac.kr/oasis) and *p* values were calculated by using log-rank (Mantel-Cox method) test ^5^.

*Motility assays using Multi-Worm Tracker:* Motility was analyzed by using Multi-Worm Tracker as previously described with modifications (Swierczek et al., 2011). Myricetin-treated worms and control worms were transferred onto plates containing FUdR at day 1 adult stage for experimental conditions consistent with lifespan assays. Eleven or twelve plates were prepared for control or myricetin conditions, and at least 15 worms were cultured on each plate. The movement of worms were recorded by using DIMIS-M (Siwon Optical Technology, Anyang, South Korea) camera for 1 min at day 1 and day 9 adult stages. Average speed of the worms were measured by the Multi-Worm Tracker software ^6^.

*Body bending assays:* Body bending assays were performed as described previously ^7^. Myricetin-treated worms and control worms were transferred onto FUdR-treated plates at day 1 adult stage for experimental conditions consistent with lifespan assays. The worms were transferred into 1 ml M9 buffer-containing wells of 24-well plates. After 30 sec of initial stabilizations, body bending of the worms was recorded by using DIMIS-M camera. The body bends of individual worms were counted for 30 sec and calculated for the number of body bends per min.

**Supplemental References**

1 Lagouge, M. *et al.* Resveratrol improves mitochondrial function and protects against metabolic disease by activating SIRT1 and PGC-1alpha. *Cell* **127**, 1109-1122, doi:10.1016/j.cell.2006.11.013 (2006).

2 Jung, H. Y. *et al.* The Korean Mistletoe (Viscum album coloratum) Extract Has an Antiobesity Effect and Protects against Hepatic Steatosis in Mice with High-Fat Diet-Induced Obesity. *Evid Based Complement Alternat Med* **2013**, 168207, doi:10.1155/2013/168207 (2013).

3 Grunz, G. *et al.* Structural features and bioavailability of four flavonoids and their implications for lifespan-extending and antioxidant actions in C. elegans. *Mech Ageing Dev* **133**, 1-10, doi:10.1016/j.mad.2011.11.005 (2012).

4 Seo, M. *et al.* RNA helicase HEL-1 promotes longevity by specifically activating DAF-16/FOXO transcription factor signaling in Caenorhabditis elegans. *Proc Natl Acad Sci U S A* **112**, E4246-4255, doi:10.1073/pnas.1505451112 (2015).

5 Yang, J. S. *et al.* OASIS: online application for the survival analysis of lifespan assays performed in aging research. *PLoS One* **6**, e23525, doi:10.1371/journal.pone.0023525 (2011).

6 Swierczek, N. A., Giles, A. C., Rankin, C. H. & Kerr, R. A. High-throughput behavioral analysis in C. elegans. *Nat Methods* **8**, 592-598, doi:10.1038/nmeth.1625 (2011).

7 Lee, D. *et al.* SREBP and MDT-15 protect C. elegans from glucose-induced accelerated aging by preventing accumulation of saturated fat. *Genes Dev* **29**, 2490-2503, doi:10.1101/gad.266304.115 (2015).

***Table S1. Analysis of serum biochemical parameters of between myricetin-treated and nontreated control mice***

| **Parameter** | **Control** | **STD** | **Myricetin** | **STD** | ***p* value vs. control** |
| --- | --- | --- | --- | --- | --- |
| Total protein (g/dl)  Albumin (g/dl)  Ala aminotransferase (mg/dL)  Asp aminotransferase (mg/dL) | 5.5  3.6  13  91 | 0.35  0.22  4.5  11.9 | 5.9  4.1  24  111 | 0.22  0.22  4.2  46 | 0.18  0.03  0.02  0.36 |
| Creatinine (mg/dL)  Glucose (mg/dL)  Triglycerides (mg/dL)  Cholesterol (mg/dL)  HDL Chol (mg/dL)  LDL Chol (mg/dL)  Uric Acid (mg/dL)  Lactate dehydrogenase (U/L)  Creatine kinase (g/L) | 0.5  93  180  122  40  5  2.2  640.9  38 | 0.0  10.8  53.5  14.4  3.5  0.0  0.57  232.5  15.7 | 0.5  102  219  132  47  5  2.2  733.8  50 | 0.0  13.8  61.7  22.5  5.7  0.0  0.27  129.1  11.7 | 0.29  0.26  0.46  0.13  1  0.48  0.29 |

**Values shown are mean (±s.e.m., n=5). P<0.05 versus control by one-tailed Student's test.**

***Table S2. Analysis of C. elegans lifespan data related to Figure 4.***

| **Strain/treatment** | **Mean lifespan ±s.e.m. (days)** | **75th percentile** | **% change** | **Number of animals that died/total** | ***p* value vs. control** | **Figure in text** |
| --- | --- | --- | --- | --- | --- | --- |
| Wild-type/control | 27.2±0.45 | 31 |  | 122/140 |  | Fig. 4A |
| Wild-type/myricetin | 31.3±0.52 | 36 | +15% | 134/140 | <0.0001 | Fig. 4A |
| Wild-type/control | 26.4±0.7 | 28 |  | 82/90 |  |  |
| Wild-type/myricetin | 33.4±1.0 | 41 | +27% | 71/90 | <0.0001 |  |
| Wild-type/control | 26.8±0.4 | 30 |  | 90/100 |  |  |
| Wild-type/myricetin | 29.5±0.6 | 34 | +10% | 90/100 | <0.0001 |  |
| Wild-type/control | 28.1±0.8 | 34 |  | 86/120 |  |  |
| Wild-type/myricetin | 32.5±0.6 | 37 | +16% | 104/120 | <0.0001 |  |
| *sir-2.1(ok434)*/  control | 26.5±0.7 | 31 | -6% | 84/120 |  | Fig. 4E |
| *sir-2.1(ok434)*/  myricetin | 25.9±0.7 | 31 | -2% | 99/120 | 0.6556 | Fig. 4E |
| *daf-16(mu86)*/  control | 16.1±0.3 | 18 | -43% | 87/120 |  | Fig. 4F |
| *daf-16(mu86)*/  myricetin | 21.4±0.5 | 24 | +33% | 92/120 | <0.0001 | Fig. 4F |
| *aak-2(ok524)*/  control | 16.5±0.6 | 18 | -41% | 60/120 |  |  |
| *aak-2(ok524)*/  myricetin | 18.9±0.5 | 24 | +15% | 68/120 | 0.0037 |  |
| Wild-type/control | 28.5±0.8 | 35 |  | 71/90 |  |  |
| Wild-type/myricetin | 33.7±0.6 | 38 | +18% | 80/120 | <0.0001 |  |
| *sir-2.1(ok434)*/  control | 25.5±0.6 | 29 | -11% | 80/90 |  |  |
| *sir-2.1(ok434)*/  myricetin | 27.3±0.7 | 32 | +7% | 96/120 | 0.0229 |  |
| *daf-16(mu86)*/  control | 17.1±0.4 | 20 | -40% | 69/90 |  |  |
| *daf-16(mu86)*/  myricetin | 22.0±0.5 | 26 | +29% | 85/120 | <0.0001 |  |
| *aak-2(ok524)*/  control | 15.4±0.5 | 20 | -46% | 71/90 |  | Fig 4G |
| *aak-2(ok524)*/  myricetin | 18.2±0.6 | 23 | +18% | 87/120 | 0.0002 | Fig 4G |

Lifespan data within the solid lines are individual experimental sets. All p values were calculated within the individual sets by using the log-rank (Mantel-Cox) method (Yang et al., 2011).

Percent (%) changes and p values for myricetin-treated worms were calculated against control worms within dashed lines in the same experimental set.

Percent (%) changes and p values for mutant worms on control treatment were calculated against wild-type worms on control treatment in the same experimental set.

**Table S3. Primer lists.**

| | ***Gene name*** | ***Accession number*** |  | ***Sequence*** | | --- | --- | --- | --- | | PGC-1α | NM_008904 | Forward | 5′-GCA TAT CGA TGC TGC TCT TTC-3′ | |  |  | Reverse | 5'-5′-GAT AAC CTG GAT CCA TAG ATC GTT-3′-3' | | PGC-1β | NM_133249 | Forward | 5′-GGA GGA GAA GGC TGT GTT-3′ | |  |  | Reverse | 5′-TAA AGG CGG CAT CCA CTC-3′ | | ERRα | NM_007953 | Forward | 5′-CAA AAC ATA TTT CTT TGT AGA GGA CAA-3′ | |  |  | Reverse | 5′5′-TTC AGC TAT TTG CTT GGG AAA-3′-3′ | | Tfam | NM_009360 | Forward | 5′-ATG TAC CCT ATG TAC CGC TTC-3′ | |  |  | Reverse | 5′-GTG TGG TGG TGG TTG GAG-3′ | | PPARα | NM_001113418 | Forward | 5′-AAT AAC ACA GAG AGA CAG ACT TG-3′ | |  |  | Reverse | 5′-CTT GGA TAC CCT TGG CTT TAG-3′ | | PPARβ/δ | NM_011145 | Forward | 5′-GTG TGT ATT GTT CCC AGT GA-3′ | |  |  | Reverse | 5′-AGT TAA TGC TAT GAG AAG ACT AAG G-3′ | | PPARγ | NM_001127330 | Forward | 5′-GGC TGC TCT CCA ATG TCA-3′ | |  |  | Reverse | 5′-CAC TCT GCG AAG ACC TCC-3′, | | UCP1 | NM_009463 | Forward | 5′-TTC CCT CCA AGA AGG ATT TG-3′ | |  |  | Reverse | 5′-ACG AGT GTA TGA GTT GTA GAA G-3′ | | UCP3 | NM_009464 | Forward | 5′-TTC CCT CCA AGA AGG ATT TG-3′ | |  |  | Reverse | 5′-ACG AGT GTA TGA GTT GTA GAA G-3 | | Sirt1 | NM_019812 | Forward | 5′-TTC CCT CCA AGA AGG ATT TG-3′ | |  |  | Reverse | 5′-ACG AGT GTA TGA GTT GTA GAA G-3′ | | Sirt3 | NM_022433 | Forward | 5′-TTC CCT CCA AGA AGG ATT TG-3′ | |  |  | Reverse | 5′-ACG AGT GTA TGA GTT GTA GAA G-3 | | Foxo1 | NM_019739 | Forward | 5′-CCT TTC CTC CTC CCT CTG -3′ | |  |  | Reverse | 5′-ACG AGT GTA TGA GTT GTA GAA G-3′ | | Cytochrome c | NM_007808 | Forward | 5′-TTC CCT CCA AGA AGG ATT TG-3′ | |  |  | Reverse | 5′-ACG AGT GTA TGA GTT GTA GAA G-3′ | | myoglobin | NM_013593 | Forward | 5′-TTC CCT CCA AGA AGG ATT TG-3′ | |  |  | Reverse | 5′-ACG AGT GTA TGA GTT GTA GAA G-3′ | | ssTnI | NM_021467 | Forward | 5′-TTC CCT CCA AGA AGG ATT TG-3′ | |  |  | Reverse | 5′-ACG AGT GTA TGA GTT GTA GAA G-3′ | | β-Actin | NM_007393 | Forward | 5′-GCG AGA AGA TGA CCC AGA T-3′ | |  |  | Reverse | 5′-ATC ACG ATG CCA GTG GTA-3′ |   PGC-1α, peroxisome proliferator-activated receptor γ coactivator1α; PGC-1β, peroxisome proliferator-activated receptor γ coactivator1β; ERR-α, Estrogen-related receptor α; Tfam, Mitochondrial transcription factor A; PPARα, peroxisome proliferator-activated receptor α; PPARβ/δ, , peroxisome proliferator-activated receptor β/δ; PPARγ, peroxisome proliferator-activated receptor γ; UCP1, uncoupling protein 1; UCP3, uncoupling protein 3; Sirt1, Sirtuin 1; Sirt3, Sirtuin 3; Foxo1, Forkhead box protein O1; ssTnI, slow skeletal troponin I |
| --- | --- | --- | --- | --- | --- | --- | --- | --- | --- | --- | --- | --- | --- | --- | --- | --- | --- | --- | --- | --- | --- | --- | --- | --- | --- | --- | --- | --- | --- | --- | --- | --- | --- | --- | --- | --- | --- | --- | --- | --- | --- | --- | --- | --- | --- | --- | --- | --- | --- | --- | --- | --- | --- | --- | --- | --- | --- | --- | --- | --- | --- | --- | --- | --- | --- | --- | --- | --- | --- | --- | --- | --- | --- | --- | --- | --- | --- | --- | --- | --- | --- | --- | --- | --- | --- | --- | --- | --- | --- | --- | --- | --- | --- | --- | --- | --- | --- | --- | --- | --- | --- | --- | --- | --- | --- | --- | --- | --- | --- | --- | --- | --- | --- | --- | --- | --- | --- | --- | --- | --- | --- | --- | --- | --- | --- | --- | --- | --- | --- | --- | --- | --- |
